# Sustainable self-healing at ultra-low temperatures in structural composites incorporating hollow vessels and heating elements

**DOI:** 10.1098/rsos.160488

**Published:** 2016-09-14

**Authors:** Yongjing Wang, Duc Truong Pham, Zhichun Zhang, Jinjun Li, Chunqian Ji, Yanju Liu, Jinsong Leng

**Affiliations:** 1 Department of Mechanical Engineering, School of Engineering, University of Birmingham, Edgbaston, Birmingham, UK; 2 Center for Composite Materials and Structures, Harbin Institute of Technology, Science Park, Harbin, People's Republic of China; 3 Department of Aerospace Science and Mechanics, Harbin Institute of Technology, Science Park, Harbin, People's Republic of China; 4 Applied Science Faculty, Delft University of Technology, Delft, The Netherlands

**Keywords:** carbon nanotubes, self-healing, fibre-reinforced composites, delamination, self-repair, smart materials

## Abstract

Self-healing composites are able to restore their properties automatically. Impressive healing efficiencies can be achieved when conditions are favourable. On the other hand, healing might not be possible under adverse circumstances such as very low ambient temperature. Here, we report a structural composite able to maintain its temperature to provide a sustainable self-healing capability—similar to that in the natural world where some animals keep a constant body temperature to allow enzymes to stay active. The composite embeds three-dimensional hollow vessels with the purpose of delivering and releasing healing agents, and a porous conductive element to provide heat internally to defrost and promote healing reactions. A healing efficiency over 100% at around −60°C was obtained. The effects of the sheets on the interlaminar and tensile properties have been investigated experimentally. The proposed technique can be implemented in a majority of extrinsic self-healing composites to enable automatic recovery at ultra-low temperatures.

## 1. Introduction

Self-healing composite materials are artificial materials that can heal after damage like living creatures do. Research efforts across the globe over the past two decades have resulted in healing efficiencies above 100%, indicating that the function or performance of the healed material can be better than that prior to damage [1]. Such healing is expected to be of great value in applications where repairing or replacing key components is challenging or even impossible, for instance, in aircraft and satellites during flight, and in equipment that is difficult to access such as offshore wind turbines.

Fibre-reinforced composites (FRCs) have gained popularity in the above-mentioned applications due to their high strength and light weight. However, the main risk in employing these materials is internal micro-cracks which may cause catastrophic failures and that are hard to detect and repair. Hence, enabling them to self-heal has been proposed as a potential method to improve the reliability of FRCs and increase their service life as well as to decrease repair costs. To enable a composite material to self-heal, capsule-based [2] and vessel-based designs [3] have been proposed. A capsule-based design involves embedding capsules containing special liquids able to cause healing of the host material. When a crack occurs, some of the capsules break and release the liquids (also known as healing agents) which fill the crack. A vessel-based design works in a similar way, but the capsules are replaced by a vascular network in one, two or three dimensions. By adopting these designs, FRCs endowed with the ability to self-heal have been created. Blaiszik *et al*. [4] and Jones *et al*. [5,6] have developed fibres functionalized with micro-capsules containing healing liquids to repair the interface between the fibres and the polymer matrix [4–6]. Moll *et al*. [7] dispersed the capsules in the matrix and reported a near 100% recovery from minor damage. Norris *et al*. [8] embedded one-dimensional hollow vessels loaded with healing agents in an FRC. Patrick *et al*. [9] invented an FRC incorporating three-dimensional vessels in a complex pattern to cope with delamination and restore mechanical properties in multiple damage cycles.

However, it is worth noting that the good healing performances reported so far are attainable only when there are favourable healing conditions, such as a suitably high ambient temperature or an appropriate radiation treatment, which is often impossible to ensure in practical applications. For instance, composites used on an aircraft may endure temperatures as low as −60°C, at which almost all healing liquids would be frozen and cannot be activated. This has become one of the main barriers to the wider adoption of self-healing composites, prompting efforts to develop systems that can self-heal regardless of environmental and damage conditions. A few researchers have been trying to do this by employing new healing liquids, and have reported healing agents able to heal at temperatures as low as 10°C [10,11]. The effects of ultra-low temperatures on a typical healing agent have also been investigated [12]. However, real high-efficiency healing at very low temperatures (−40 to −80°C) is still impossible.

Here, we report a design to enable self-healing in FRCs at ultra-low temperatures. With the proposed approach, healing was fulfilled by two components: three-dimensional vessels and a thin layer of conductive material. The vessels were embedded inside the structural composites with the purpose of delivering and releasing healing agents. The thin layer was to supply heat internally from the composites to cause de-icing and provide a suitable temperature for healing. We selected a vessel-based design instead of a capsule-based design because the former is capable of recovery from large-area damage, as healing agents can be continuously pumped into the vessels [3,9,13–18]. Furthermore, the vessel network would in general only have minor effects on the tensile properties of the composites [19]. The fabricated composites are able to recover from severe delamination with average efficiencies around 100% at ultra-low temperatures. We also discuss the effects of the conductive sheets on interlaminar and tensile properties of the laminates. Experimental results indicate that the sheets reduced interlaminar strength but increased tensile properties.

## 2. Material and methods

### 2.1. Structure of the composite

**Figure 1.**
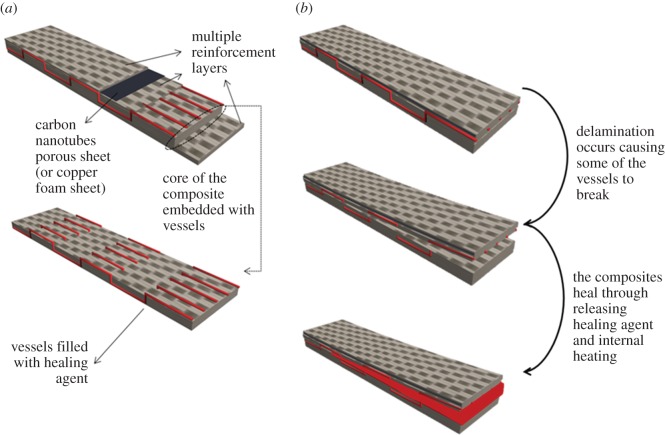
(*a*) Internal structure of the composites and (*b*) damage–bleeding–healing process.

The composite is a glass fibre-reinforced laminate embedded with wave-like hollow vessels and conductive sheets, as shown in figure 1*a*. When delamination occurs at low temperature, cracks can propagate and break the vessels. Heat could be generated internally through electrical heating to defrost, releasing liquid healing agents into the cracks. Solidification of the agents could also be accelerated by the internal heat. The healing process is shown in figure 1*b*. Here, we discuss the wave-like hollow vessels and conductive sheets separately.

#### 2.1.1. Wave-like hollow vessels in the core

The wave-like configuration was proposed by Patrick *et al*. [9] who were the first to use it as well as a herringbone configuration for self-healing in FRCs. The hollow vessels can be made by the vapourization of sacrificial components (VaSC) incorporated in the material with the reinforcement fibres. Poly(lactic acid) (PLA) sacrificial fibres 300 µm in diameter were selected as sacrificial components as they left little residue, greatly reducing the risk of blocking the channels [9,20–22]. Other methods to fabricate internal hollow structures include using hollow fibres [23,24], electrostatic discharging [25] and laser direct-writing [26]. However, only VaSC can produce large-scale three-dimensional vascular networks following an accurate pre-designed pattern.

#### 2.1.2. Conductive porous sheets

**Figure 2.**
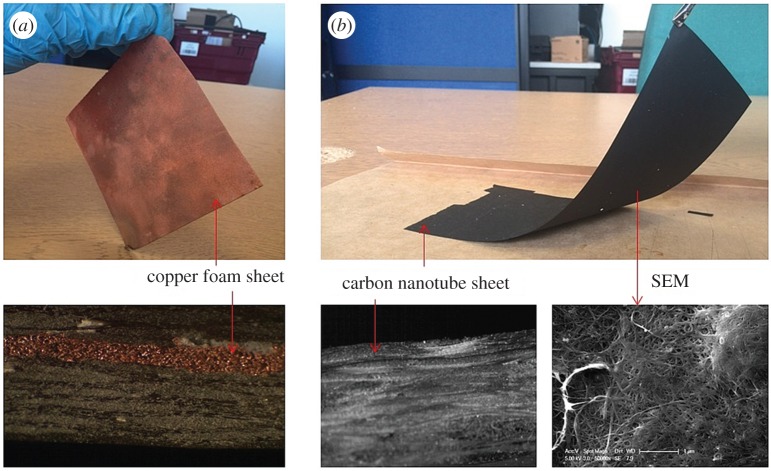
(*a*) Porous copper foam sheet and (*b*) porous carbon nanotube sheet.

The conductive layer must satisfy two requirements: good electrical conductivity and good thermal conductivity. To fabricate this conductive layer, two types of conductive sheet were selected: porous copper foam sheet (CFS) and porous carbon nanotube sheet (CNS). Metal foam has already been applied in batteries [27], heat exchange devices [28] and energy absorbers [29]. The CFS (figure 2*a*) had a thickness of 0.5 mm and a porosity of 96 ∼ 98%. It had a thermal conductivity around 10 W m^−1^ K^−1^, very high electrical conductivity (approx. 3.9 × 10^6^ S m^−1^) and a large area of contact with the host material. The other conductive sheet (figure 2*b*), CNS, with a thickness of 40 µm, had a stable electrical conductivity of 1.25 ∼ 1.38 × 10^−4^ Ω m and a thermal conductivity estimated to be in the range 100 ∼ 1000 W m^−1^ K^−1^. The thermal conductivity was measured by using the method in the work of Wang *et al*. [30]. The CNS was fabricated by multiple steps of single wall carbon nanotubes (CNTs) dispersion and suspension filtration [31].

### 2.2. Fabrication procedure

VaSC and resin infusion were used to make FRCs incorporating hollow vessels and heating components. The PLA sacrificial fibres (300 µm VascTech fibres, CU Aerospace Ltd.) were manually embedded into eight layers of woven glass fibres (area density of 290 g m^−2^ for each layer) in a square-wave-like configuration. The incorporated reinforcement fibres and other untreated reinforcement fibres, as well as the conductive sheets, were deposited layer by layer in the following sequence: bottom—four layers of normal glass fibres—eight layers of glass fibres with the sacrificial components—two layers of normal glass fibres—conductive sheet—two layers of normal glass fibres—top. A nylon sheet was placed at the mid-plane position and offset 30 mm from one of the edges. The sheet served as a crack created during the fabrication of the composite. Epoxy resin and hardener (very high temperature epoxy, Easy Composites Ltd.) were mixed at a ratio of 100 : 35 parts by weight and degassed at 35°C for 30 min in a vacuum chamber. Afterwards, a resin infusion process took place to make the composites. The mixture was cured for 36 h at room temperature and then put through post-cure heating cycles at 40°C, 60°C, 80°C, 100°C and 120°C each for 1 h, and 140°C for 3 h, as suggested by the supplier. After the resin was fully cured, the composite which had a thickness of 4 mm was cut into 180 × 25 mm pieces using a grit saw and polished with sand paper. The cross section of the samples is shown in figure 2*a* (FRCs + CFS) and 2*b* (FRCs + CNS).

The sacrificial components were removed by placing the samples in a 200°C vacuum chamber for 24 h. After the fabrication of the hollow vessels, the healing agent, which was a pre-mixed two-part epoxy (RT151, ResinTech Ltd.) dyed in red, was injected into the vessels using a controllable liquid dispenser. The complete fabrication procedure is detailed in the electronic supplementary material.

### 2.3. Healing performance assessment and analysis

**Figure 3.**
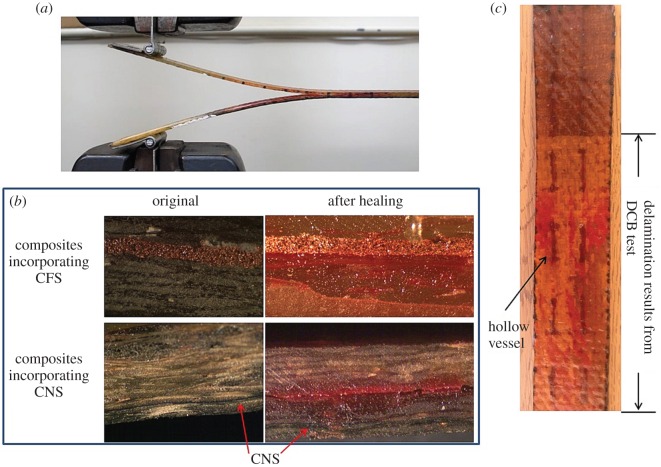
(*a*) DCB test of a healed composite specimen incorporating CNS, (*b*) composites incorporating CNS and CFS and (*c*) fibre-reinforced composite incorporating wave-like micro vessels after DCB test.

The mechanical strength of the samples was assessed using the double cantilever beam (DCB) test, as shown in figure 3*a*, following the procedure described in §2.5. For each sample, a total of three DCB tests were conducted. The first test was to measure the original interlaminar strength and to break the sample. Afterwards, the broken sample was loaded again for the second test to reveal the residual strength. Then it was given a 24 h rest to heal at −60°C before taking the third test to assess the healing performance. The healing process started with a continuous injection of healing agents into the vessels, followed by the specimens being placed in an ultra-low temperature (−60°C) chamber. During healing, the conductive layer was electrically heated. The power used was 7 W and 9.45 W for CFS and CNS, respectively. A thermometer was attached to the specimen to monitor its temperature. The cross section of the healed sample is shown in figure 3*b*. During the test, cracks propagated and traversed the wave-like vessels as in figure 3*c*.

#### 2.3.1. Calculation of healing efficiency

The healing efficiency in relation to peak load (*η*_1_) was calculated as [9,32]:

2.1 $$\eta_{1}=\frac{L_{\mathrm{Healed}}}{L_{\mathrm{Virgin}}}\times 100\%,$$

where *L*_Virgin_ is the achieved maximum load before damage and *L*_Healed_ is the maximum load after the specimen has recovered from an 80 mm mode-I fracture.

The healing efficiency in relation to fracture energy (*η*_2_) was calculated as [9]:

2.2 $$\eta_{2}=\frac{U_{I,\mathrm{Healed}}}{U_{I,\mathrm{Virgin}}}\times 100\%,$$

where *U*_I,Healed_ and *U*_I,Virgin_ are the fracture energies of a specimen before damage and after recovery, respectively, derived from first principles as the area under the load–displacement trace at a particular crack length.

### 2.4. Effects of the conductive sheets on interlaminar properties

The introduction of a conductive sheet created a new layer inside the laminates. The effects of having this layer on interlaminar properties were revealed using DCB tests to compare samples with and without the sheets.

Three groups of samples, each containing five specimens, were made using the techniques described in §2.2. In the first group, specimens were fibre-reinforced laminates with the CNS in the middle layer. In the second group, the CNS was replaced by the CFS. Specimens in the third group were ordinary fibre-reinforced laminates. All specimens were 120 mm in length, 20 mm in width and 4 mm in thickness, and had a 20 mm deep pre-crack on one edge. DCB tests followed the procedure outlined in §2.5.

### 2.5. Double cantilever beam test

Aluminium piano hinges were attached onto the specimen using a structural adhesive (Loctite 330 Glue and Activator Multibond Kit). After heating for 6 h at 60°C, the adhesive was fully cured and testing was carried out on an MTS Criterion Model 43 machine. The specimens were loaded through the bonded hinges in quasi-static tension to induce mode-I fracture propagation along the mid-ply interlaminar region until the crack reached 80 mm. The displacement-controlled crosshead speed was 5 mm min^−1^ during loading.

### 2.6. Effects of the conductive sheets on tensile properties

**Figure 4.**
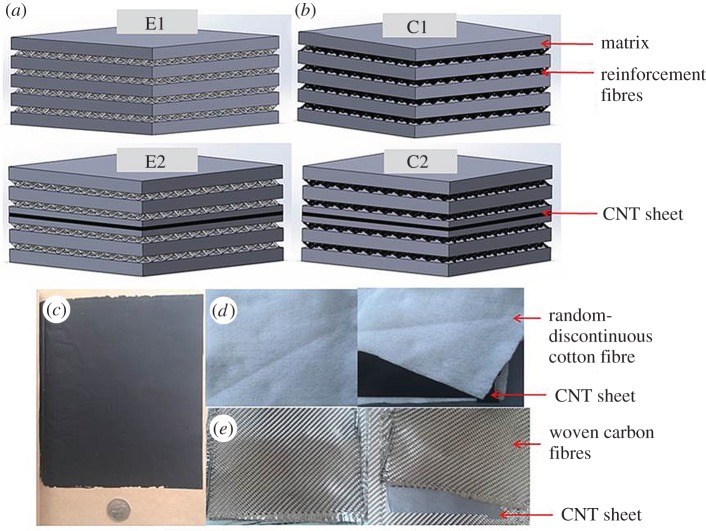
(*a*) Schematic of composite E, (*b*) schematic of composite C, (*c*) CNT porous sheet, (*d*) random-discontinuous cotton fibres and CNT sheet and (*e*) woven carbon fibres and CNT sheet.

The effects on two types of composites were investigated: random-discontinuous cotton-FRCs (type E) and woven carbon-FRCs (type C) (figure 4). E contained four layers of cotton breather cloth and C had four layers of woven carbon fibres. As the fillers are different, E and C have different bearing strengths, representing weak and strong polymer composites, respectively.

E and C are further divided into two subgroups (E1/E2 and C1/C2) as shown in table 1. The effects of the CNT layer can be revealed through comparing E1 (or C1) with E2 (or C2).

**Table 1.** Details of the subgroups of composites.

subgroup code	description	no. specimens
E1	random-discontinuous cotton fibre composites	5
E2	random-discontinuous cotton fibre composites embedded with porous CNT layer	5
C1	woven carbon fibre composites	5
C2	woven carbon fibre composites embedded with porous CNT layer	5

The porous CNT layer was fabricated inside E2 and C2 by embedding a CNS using the techniques described in §2.2. After the resin was fully cured, the composites were cut into 100 × 9 × 2 mm specimens. The specimens were placed in a chamber at 50°C for 12 h to release internal strain. A MTS Criterion testing machine (MTS Criterion Model 43 Electromechanical Universal Test System) was used to collect data. Each specimen was subject to tension parallel to its long edge whose effective length is 15 mm. The loading rate was 0.5 mm min^−1^. After tensile testing, the samples were observed under optical and electron microscopy to reveal cracking patterns and the nature of the fracture.

## 3. Results and discussion

### 3.1. De-icing performance

**Figure 5.**
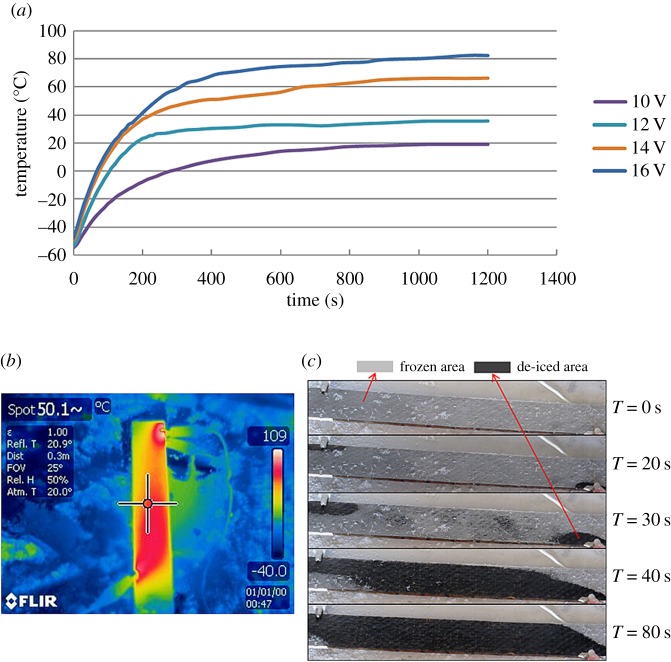
(*a*) Temperature of the specimen as a function of time and applied voltage, (*b*) thermal distribution in the composite heated by CNS in an ultra-low temperature chamber and (*c*) de-icing of composite with CNS.

Figure 5*a* shows the temperature of a sample incorporating CNS under electrical heating. With the applied voltage maintained between 10 and 16 V, the steady-state temperatures of the specimens remained in the range 20–85°C (figure 5*a*), which was sufficiently high to enable curing in 24 h (figure 5*b*). There was no severe heat concentration due to the use of a relatively low electrical current (200 mA) and its good thermal conductivity. The composite was able to be fully de-iced in 90 s (figure 5*c*), which also demonstrates the efficiency of CNS.

The composite with CFS was able to keep the temperature in the range 5–20°C. However, for the design using CFS, it was found that although the healing agent was still active in the temperature range, 24 h was not long enough for it to cure fully. Increasing the electrical power to raise the internal temperature may seem a solution to this problem. However, this was almost impossible as the copper foam had an extremely low resistivity, and so a very large electrical current—55 A in this case—was required to generate the heat necessary to keep the healing agent active. Such a high electrical current might generate extremely high temperature at the points of contact between the sample and the power supply and may cause local melting. In our experiments, although four of the five samples tested successfully healed themselves, there was still one that failed due to heat concentration. Therefore, although increasing the supplied power will raise the internal temperature, it also heightens the risk of local overheating as a side effect.

### 3.2. Healing performance

**Figure 6.**
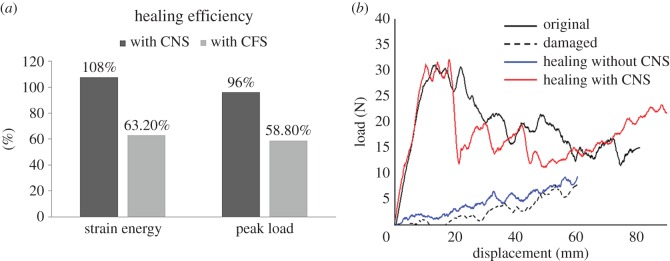
(*a*) Healing efficiency of composite with CNS and CFS and (*b*) displacement–load curve for a typical sample incorporating CNS.

After 24 h of healing at −60°C, the recovered mechanical properties of the samples are given in tables 2 and 3 and figure 6*a*,*b*.

**Table 2.** DCB test results quantifying healing performances.

specimen no.	original peak load (N)	recovered peak load (N)	original fracture energy (N mm)	recovered fracture energy (N mm)	healing efficiency for peak load (%)	healing efficiency for fracture energy (%)
GFRC + CNS						
1	31.5	32.5	1544.2	2077.4	103	135
2	30	29.8	1437.5	728.8	99.30	50.70
3	31.2	26.6	1356.8	1329	85.30	98
4	28	26.44	1586.5	2238	99.40	141
5	32.3	30.4	1565.35	1783.5	94.10	114
GFRC + CFS						
1	65	40	3484.8	3298.4	61.50	94.70
2	62	45	2978.6	1837.3	72.60	61.70
3	58	30	3610.2	1141.7	51.70	31.60
4	51	55	2564.3	3284.1	108	128
5	55	n.a.	3024.5	n.a.	0	0

**Table 3.** Healing efficiency summary.

	no. samples	max. healing efficiency for fracture energy (%)	min. healing efficiency for fracture energy (%)	ave. healing efficiency for fracture energy (%)	max. healing efficiency for peak load (%)	min. healing efficiency for peak load (%)	ave. healing efficiency for peak load (%)
GFRC + CNS	5	141	50.7	107.7	103	85.3	96.22
GFRC + CFS	5	128	0	63.2	108	0	58.8

For the composite with CNS, an average healing efficiency of 107.7% for fracture energy and 96.22% for peak load was achieved. The maximum healing efficiency for fracture energy was 141%.

Four samples of the composites with CFS, out of five, successfully recovered from damage with an average healing efficiency of 63.2% for fracture energy and 58.8% for peak load. The maximum healing efficiency for fracture energy was 128%.

The results indicate that the composite materials with either CFS or CNS are able to self-heal at ultra-low temperatures. Furthermore, CNS is the better of the two types of conductive layer. The electrical resistivity of CFS was too low and therefore a very high electrical current was required to generate heat, causing potential overheating for everything connected in series with the composite, especially at the contact points. Such a high current may locally generate temperatures approaching 500°C. Also, the thermal conductivity of CFS is not high enough to allow heat transfer to other areas. This caused parts of the composite to be damaged by heat concentration, while the rest remained so cold that the healing agents could not function properly. As a result, the composite only achieved an average healing efficiency no greater than 70%. CNS, on the other hand, had an electrical resistivity which was much higher than that of CFS. Thus, only a small electrical current was sufficient to generate the required heat. The thermal conductivity was also sufficiently high to enable heat to flow rather than concentrate at local hot spots.

To achieve satisfactory healing performances, another important factor was how well the healing agent covers the composite materials. There have been concerns that the wave-like design in which the vessels penetrate multiple layers cannot ensure a large coverage and might be less effective than that adopted in straight hollow-fibre-based self-healing composites [24,33,34]. However, the high healing efficiency proved that a large coverage was achievable. Another ongoing study by the authors has shown that having a large coverage of vessels in multiple layers is more important than having extensive interlaminar coverage [35].

It might be possible to achieve self-healing at ultra-low temperatures using new healing agents. However, it is worth noting that any healing agent will have an active temperature range, outside of which the healing process cannot take place. All established healing agents so far have narrow active temperature ranges, and none of them is suitable when a high healing efficiency over a wide temperature range, such as −60 to 100°C, is required. Even if a new healing agent can be developed for that temperature range, it would become ineffective as soon as the operating temperature fell outside the range. Thus, the development of new healing agents is not an effective way to achieve sustainable self-healing. The essence of our approach is that there is no limitation relating to types of healing agents and composites—all established composites that self-heal in certain temperature ranges can be modified based on the design presented here to achieve self-healing at ultra-low temperatures. By tuning the power provided to the conductive layer, the internal temperature of composites can be altered regardless of the ambient temperature to provide suitable conditions for any healing agent.

### 3.3. Effects of conductive sheets on interlaminar properties

**Figure 7.**
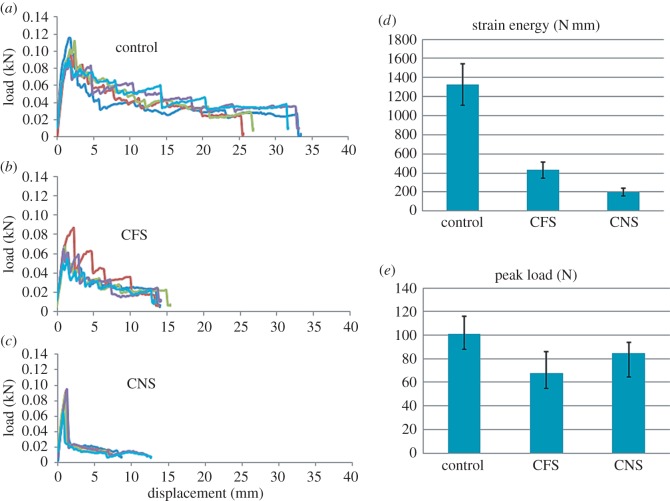
Mode I load versus displacement curve for the laminates with (*a*) no embedment, (*b*) a copper foam sheet and (*c*) a carbon nanotube sheet. Results of DCB testing: (*d*) fracture energy and (*e*) peak load.

The mode I load versus displacement curves for composites with and without conductive sheets are shown in figure 7*a*–*c*. Each figure displays the load–displacement curves for five tested specimens. Comparison of composites with and without conductive sheets (figure 7*d,e*) indicates obvious reductions in fracture energy and peak loads. The adoption of a porous sheet was to form a short-fibre-reinforced layer inside the composite so that the layer could bond strongly to the host material. The results might be explained by the possibility that the resin had not fully infiltrated the porous layer and that the conductive sheet should have a very rough surface to strengthen the interface bonding with the host material. A potential solution might be to optimize the surface pattern of the sheet to help preserve the interlaminar strength while ensuring heating efficiency. Techniques similar to those used with vascular networks for vessel-based self-healing materials could be adopted as the same potential issue with delamination exists there also [36–38].

### 3.4. Effects of carbon nanotube sheets on tensile properties

**Figure 8.**
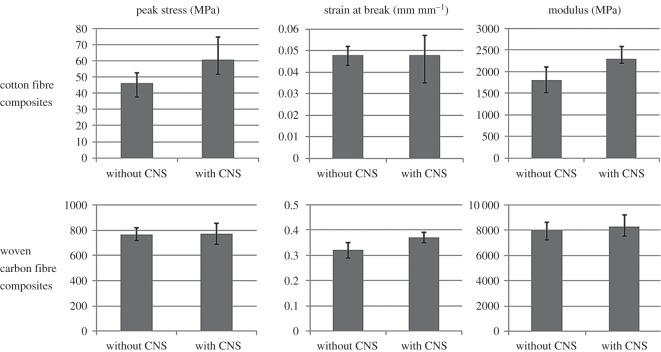
Tensile properties of composites with and without carbon nanotube sheets.

**Figure 9.**
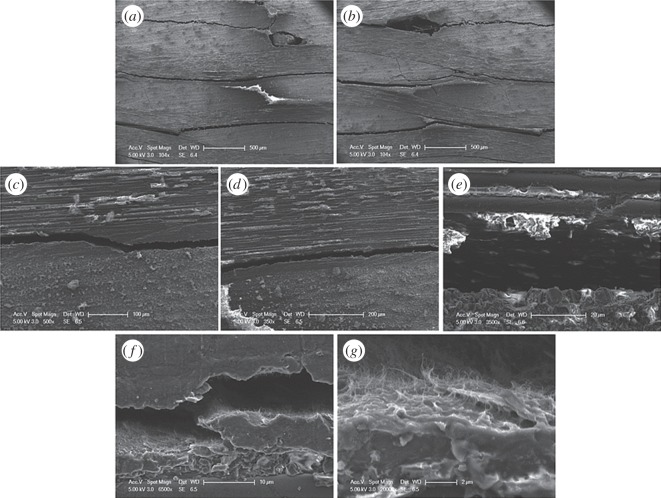
Scanning electron microscopy (SEM) images of carbon fibre composite incorporating carbon nanotube sheets after tensile tests. SEM image of crack in carbon fibre-reinforced composite incorporating carbon nanotube sheet. (*a*,*b*) Cross section of a damaged sample, (*c*–*e*) delamination of carbon fibres and matrix, (*f*,*g*) delamination of carbon nanotube sheet and matrix.

The tensile properties of composites with and without the carbon nanotube sheets are shown in figure 8. The experimental results indicate that CNTs can significantly improve the tensile strength of polymer composites without much affecting their elasticity modulus. For cotton fibre composites, failure happened with fracture of both the CNT sheet and the surrounding material and there was no obvious delamination. Considering that the CNT sheet was the primary loading component in cotton fibre composites, it can be expected that they failed once the sheet had reached its ultimate strain and ruptured. Unlike in cotton fibre composites, failure in carbon fibre composites was the result of a combination of rupture of the sheet, breaking of reinforcement fibres and delamination, as shown in figure 9. However, in a carbon fibre composite material, delamination always needs to propagate through the matrix between two adjacent carbon fibre layers. When a layer of CNTs is present, a crack would need to penetrate it before reaching the next carbon fibre layer and this provides extra resistance to crack propagation and delamination. This could be the reason why the introduction of CNT sheets improved the tensile properties of carbon fibre composites.

## 4. Conclusion

We have shown that self-healing at ultra-low temperatures can be implemented by adding vessels and a porous conductive layer into a composite material. Healing agents were continuously injected into the vessels and were released after the host material was damaged. The conductive layer increased the temperature of the composites through electrical heating to assist the flow and curing of healing agents. Both the CFS and the CNS were able to act as conductive layers. However, the composite with the CNS was able to self-heal more effectively and stably. As a result, healing in FRCs at a temperature around −60°C was achieved with an average recovery of 107.7% in fracture energy and 96.22% in peak load. The effects of the conductive sheet on interlaminar properties and tensile properties were experimentally investigated. It was found that the introduction of a CNS increased the tensile strength of polymer composites, but had negative effects on interlaminar properties. A potential solution might be to include patterns on the sheet surface to preserve interlaminar properties without significantly affecting heating performance.

## Supplementary Material

Supporting information

## Data accessibility

The methods and materials used in this work are described in the body of the manuscript and the electronic supplementary material.
