# Supplementary material for: Sustainable self-healing at ultra-low temperatures in structural composites incorporating hollow vessels and heating elements
Source: R Soc Open Sci. 2016 Sep 14;3(9):160488. doi: 10.1098/rsos.160488 (PMC5043331; doi:10.1098/rsos.160488)
Supplement: Supporting information [file rsos160488supp1.docx]

Supporting information

The fabrication of samples

1. **Fabrication of the core of the laminates**. The core of the laminates (eight layers of reinforcement fibres in the middle) was incorporated with PLA sacrificial fibre in a wave-like configuration by needle stitching. To install the PLA sacrificial fibres, eight layers of woven glass fibres (area density of 290 g/m2 for each layer) were fixed on a needlework frame to prevent the distortion of the woven architecture. The PLA sacrificial fibres (300µm VascTech fibres, CU Aerospace Ltd.) were manually embedded into the layers of woven glass fibres in a square-wave-like configuration.
2. **Resin Infusion preparation**. A polypropylene table ready for resin infusion was covered with PVA releasing agent to prevent damage during de-moulding. The releasing agent became completely dry in 30 min at room temperature. The incorporated reinforcement fibres and other untreated reinforcement fibres as well as the conductive sheets were deposited layer by layer in the following sequence: Bottom – Four layers of normal glass fibres – Eight layers of glass fibres with the sacrificial components – two layers of normal glass fibres – conductive sheet – two layers of normal glass fibres – Top. A nylon sheet was placed at the mid-plane position and offset 30 mm from one of the edges. The sheet served as a crack created during the fabrication of the composite. The laminates were covered with a peel ply, resin infusion mesh and infusion spiral before sealing with sealant tape and vacuum bagging film.
3. **Resin infusion**. Air was extracted from the sealed space by using a vacuum pump and a PVC vacuum hose to reach a vacuum level of 17 kPa. After the vacuum pump was turned off, the sealed space was left for 1 hour to ensure no pressure drop. Epoxy resin and hardener (Very High Temperature Epoxy, Easy Composites Ltd.) were mixed at a ratio of 100:35 parts by weight and degassed at 35 ⁰C for 30 minutes in a vacuum chamber. Afterwards, the mixture was infused into the sealed space.
4. **Curing.** After infusion, the mixture was cured for 36 h at room temperature and then put through post-cure heating cycles at 40 ⁰C, 60 ⁰C, 80 ⁰C, 100 ⁰C and 120 ⁰C each for one hour, and 140 ⁰C for 3 hours, as suggested by the supplier.
5. **Cutting.** After the resin was fully cured, the composite was cut into identical specimens. The fully cured composite which had a thickness of 4 mm was cut into 180 mm × 25 mm pieces using a grit saw and polished with sand paper. The cutting and polishing followed a high-speed-low-force strategy to avoid damage. The cross-section of the samples are shown in **Figs. 2a** (FRCs + CFS) and **2b** (FRCs + CNS).
6. **VaSC.** The specimens were heated up to 200 ⁰C in the vacuum chamber for 24h to remove the sacrificial components. This left a hollow vascular network inside the composite in a wave-like configuration.
7. **Injection of healing agents.** After the fabrication of the hollow vessels, the healing agent, which was a pre-mixed two-part epoxy (RT151, ResinTech Ltd.) dyed in red, was injected into the vessels with a controllable liquid dispenser.
